# A Forward Genetic Approach in *Chlamydomonas reinhardtii* as a Strategy for Exploring Starch Catabolism

**DOI:** 10.1371/journal.pone.0074763

**Published:** 2013-09-03

**Authors:** Hande Tunçay, Justin Findinier, Thierry Duchêne, Virginie Cogez, Charlotte Cousin, Gilles Peltier, Steven G. Ball, David Dauvillée

**Affiliations:** 1 Unité de Glycobiologie Structurale et Fonctionnelle UMR 8576, CNRS- Université des Sciences et Technologies de Lille, Villeneuve d'Ascq, France; 2 Institute Micalis UMR1319, INRA, Thiverval-Grignon, France; 3 UMR Biologie Végétale et Microbiologie Environnementale, Laboratoire de Bioénergétique et Biotechnologie des Bactéries et Microalgues, CEA-CNRS- Aix Marseille Université, Saint-Paul-lez-Durance, France; Instituto de Biología Molecular y Celular de Plantas, Spain

## Abstract

A screen was recently developed to study the mobilization of starch in the unicellular green alga *Chlamydomonas reinhardtii*. This screen relies on starch synthesis accumulation during nitrogen starvation followed by the supply of nitrogen and the switch to darkness. Hence multiple regulatory networks including those of nutrient starvation, cell cycle control and light to dark transitions are likely to impact the recovery of mutant candidates. In this paper we monitor the specificity of this mutant screen by characterizing the nature of the genes disrupted in the selected mutants. We show that one third of the mutants consisted of strains mutated in genes previously reported to be of paramount importance in starch catabolism such as those encoding β-amylases, the maltose export protein, and branching enzyme I. The other mutants were defective for previously uncharacterized functions some of which are likely to define novel proteins affecting starch mobilization in green algae.

## Introduction

Starch represents the most widespread storage polysaccharide found in plants and algae. This insoluble polymer of glucose not only defines an essential source of calories for human and animal nutrition but is also used in many non-food applications including but not restricted to biofuel and hydrogen production. Starch is made of two distinct subfractions: the minor amylose fraction is synthesized by a starch granule bound isoform of starch synthase called GBSS (Granule Bound Starch Synthase). Amylopectin, the major fraction, confers to starch most of its physical properties and in particular its insoluble semi-crystalline nature. Even if amylopectin and glycogen share the same primary structure (they are both composed of linear glucose chains linked together through α-1,4 O-glycosidic bonds and branched in α-1,6), the synthesis of these two storage polysaccharides requires a completely different set of enzymes. While bacterial glycogen is synthesized and degraded through the use in most cases of no more than 5–6 activities [1] starch metabolism in green algae and land plants involves more than 30 different enzymes [2]. The high complexity of this metabolic pathway in the green lineage is conserved from the tiniest free-living green alga known to date to maize [2]. Starch is found in the plastids of both photosynthetic and nonphotosynthetic cells. In source organs as leaves it is accumulated during the light period and degraded in the dark to provide substrates for leaf respiration and to supply the plant with sucrose. Transient starch turnover is crucial for normal plant growth as revealed by the reduced growth rates of mutant plants unable to synthesize or breakdown the polysaccharide [3]. Storage starch found in amyloplasts of stems, roots, seeds and tubers is remobilized to support plant development as seedling establishment after germination [4] or in response to high demand for carbon as for nectar secretion [5].

The starch biosynthetic process has been extensively studied thanks to both reverse and forward genetics approaches. The high number of enzymatic activities involved and the interaction found between them in the same protein complexes [6], [7] reveal the high complexity of the anabolic pathway needed to achieve amylopectin biogenesis. It is therefore expected that mobilization of such a molecule would also require a complex catabolic pathway.

Significant progress in our understanding of starch mobilisation has been made mostly in the last decade. It now defines a very active domain of research that essentially concerns diurnal Arabidopsis leaf starch mobilisation (for review, see [8]). One of the most remarkable discoveries consists of the involvement of dikinases as essential actors of starch degradation. These enzymes, glucan water dikinase (GWD; [9]) and phosphoglucan water dikinase (PWD; [10]) were first found as minor proteins bound to starch granules and were subsequently demonstrated to phosphorylate glucose residues of amylopectin crystalline sections during starch breakdown. However, the exact link between this phosphorylation process and the ability displayed by degradation enzymes to initiate polysaccharide mobilization is not yet fully understood. It has been recently proposed that this phosphorylation opens up the crystalline structure thereby facilitating subsequent hydrolysis [11]. The process is even more complex as specific phosphatases called Sex4 [12], and LSF2 [13] are necessary to achieve efficient starch degradation. Another homologous protein called LSF1 [14] is also necessary for efficient starch breakdown but this protein was not yet proved to contain a phosphatase activity and may act as a regulator. In addition to the initial discovery of GWD, the analyses performed on different T-DNA insertion mutants of *Arabidopsis thaliana* have yielded a working model for the recurring starch degradation occurring in leaves of this model organism [15]. Taken together, the data revealed the importance of exo-amylases (β-amylases; [16]) and a specific type of isoamylase (ISA3; [17]) as major actors of the starch degradation process in Arabidopsis leaves at night. Briefly, the starch granule is subjected to phosphorylation/dephosphorylation thereby allowing the action of hydrolytic activities. The maltose produced by β-amylases is then exported to the cytosol via a plastidial maltose transporter MEX1 [18]. In this compartment, a transglucosylation reaction occurs allowing the release of glucose (catalysed by DPE2, [19]). Nevertheless, the catabolic model established in Arabidopsis may not represent a consensus of starch mobilization in photosynthetic tissues. It may predominantly reveal a pathway that is active in only a very specific subset of physiological conditions. These may be limited to massive and immediate export of carbon in the leaf cytosol to feed the nonphotosynthetic parts of the plant at night. Moreover, even if reverse genetics was extensively used to raise this model, the most surprising steps were revealed through distinct approaches such as biochemistry for the initial dikinase discovery or forward genetics for the Mex maltose transporter. While α-amylase activities were thought not to participate to starch degradation [20] in Arabidopsis leaves, a recent study revealed that the degradation initiated by the glucan phosphorylation process would require the action of three hydrolytic activities including one particular isoform of α-amylase called AMY3 [21]. Hence there is room to better our knowledge of starch catabolism and to expand these studies to organisms which are not only amenable to very productive forward genetic approaches but which in addition would afford for a greater variety of physiological conditions not restricted to the massive carbon export displayed by Arabidopsis leaf cells. We therefore believe that the unicellular green alga *Chlamydomonas reinhardtii* which has been by the past intensively used to study starch synthesis [22], [23], [24], [25], [26], [27] offers a very useful model to deepen our knowledge of starch mobilization. In addition to offering an excellent forward genetics system with straightforward mutant screening procedure, Chlamydomonas was also shown to offer a very diverse spectrum of relevant physiological growth conditions [28] thereby mimicking source sink and storage tissues of higher plants to study the impact of diverse mutations.

In this work, we report the production and the preliminary characterization of an insertional mutant bank of the green alga model *Chlamydomonas reinhardtii*. Our goal was to assess the suitability of the phenotypic screening procedure we set up in the green alga model [29] and that was recently used to study the link between starch degradation and hydrogen production [30]. The molecular analyses performed on our mutants allowed us to identify mutations either in enzymes involved in starch degradation *per se* or in putative regulatory functions revealing the importance to combine both reverse and forward genetic approaches to obtain a clear view on starch catabolism in plants and algae. Our data obtained so far offer valuable information for the community involved in the study of carbon metabolism in plants and reveal potential functions which may define suitable targets to control the catabolic process for bioenergy production purposes. They also not only validate the two step iodine screening procedure as a tool in identifying starch catabolic functions but also reveal the complexity of the physiological processes taking place in the cells as non directly relevant functions may have been identified too. The complete characterization of the most promising mutants including functional complementation and genetic cosegregation analyses will be described elsewhere.

## Results

### Generation of the insertional mutant libraries and phenotypic screening

Two independent insertional libraries were generated by transforming our *Chlamydomonas reinhardtii* wild-type reference strain 137C with either the *EcoRI* linearized pSL18 plasmid [31] or with a 1,9 kb PCR product corresponding to the paromomycin cassette (see Methods). Prior to transformation the cell pellets were treated with home-made autolysin to allow the transformation with glass beads. The efficiency of transformation was clearly different as we obtained around 2000 paromomycin resistant clones with 1 µg of linearized plasmid while less than 100 were observed with the same quantity of PCR products. This difference could be attributed to the limited size of the non-essential region of the foreign DNA which can be used during the integration process and that most of the insertions with PCR products did not confer paromomycin resistance to the algae. With this approach, we generated 3,000 and 13,000 mutants with respectively the PCR cassette and the linearized plasmid which were thereafter screened with iodine to detect the strains defective for starch catabolism.

**Figure 1:**
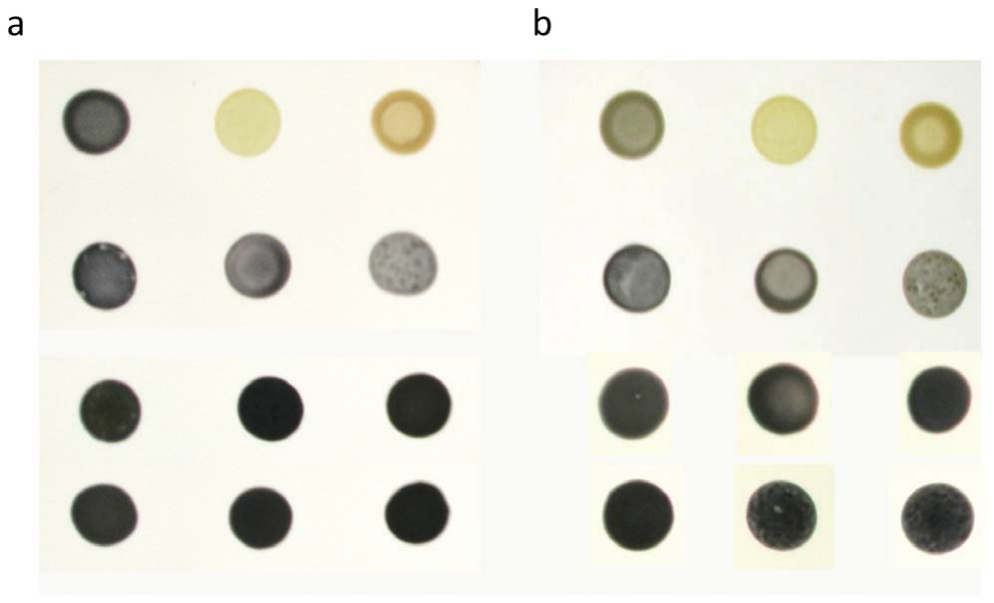
Two step iodine screen of putative starch catabolic mutants. Cell patches of the wild-type strain 137C, two mutant strains defective for starch biosynthesis and of the three mutant classes are displayed after staining with iodine vapors. The cells were incubated 5 days under nitrogen starvation in the light (a) or one more day in the dark after the removal of the starvation (b). The wild-type reference 137C, the BafJ4 mutant strain lacking starch and the BafR1 mutant producing only amylopectin are shown at the top while the CAT1, 6, and 9 (second lane); the CAT17, 21, and 23 (third lane); the CAT 3, 19, and 22 (fourth lane) represent respectively mutants of the three classes.

The natural interaction between iodine and α-1,4-linked-glucans has been used extensively to study starch anabolism in Chlamydomonas [26]. However, as a Chlamydomonas strain contains enough starch to appear black while subjected to iodine vapors, the screening procedure has to be modified in order to detect strains which are potentially containing more polysaccharide than a wild-type. Two successive steps were then used to detect the strains in which the insertion has modified either the amount of polysaccharide or produce a defect in the rate of starch mobilization. In a first step, cells were spotted in duplicate on nitrogen free TAP plates and incubated for 5 days under continuous light to trigger massive starch deposition. For one of the plates, the starvation was removed after this incubation by applying 10 µL of 0,15 M ammonium chloride and incubated in total darkness for one more day. Both plates were then subjected to iodine vapors and immediately photographed. During the incubation in the dark, a strain competent for starch mobilization will be able to degrade nearly all its storage polysaccharide and will then loose its black stain with iodine (Figure 1). A strain which is defective for starch degradation or which contained much more polysaccharide than a wild-type before the transition to darkness will remain black revealing the presence of residual starch. The figure 1 is illustrating the phenotype of the wild-type strain 137C and of 9 mutants that have been selected as putative starch catabolic mutants after a confirmation round of screening. In total, we selected 31 strains displaying a clear black phenotype after 24 h of degradation (that we named CAT1 to CAT31) from our plasmid insertional mutagenesis procedures and 7 generated with the PCR product (called CAT32 to CAT38). The same ratio of starch catabolic mutant was then observed for both foreign DNA used in this study (1 mutant for 428 resistant clones with the PCR product and 1 for 483 with the linearized plasmid). All these mutants were then subjected to biochemical characterizations in order to determine which of the candidates identify *bona fide* starch catabolism mutants

### Biochemical characterizations of the CAT mutants

**Figure 2:**
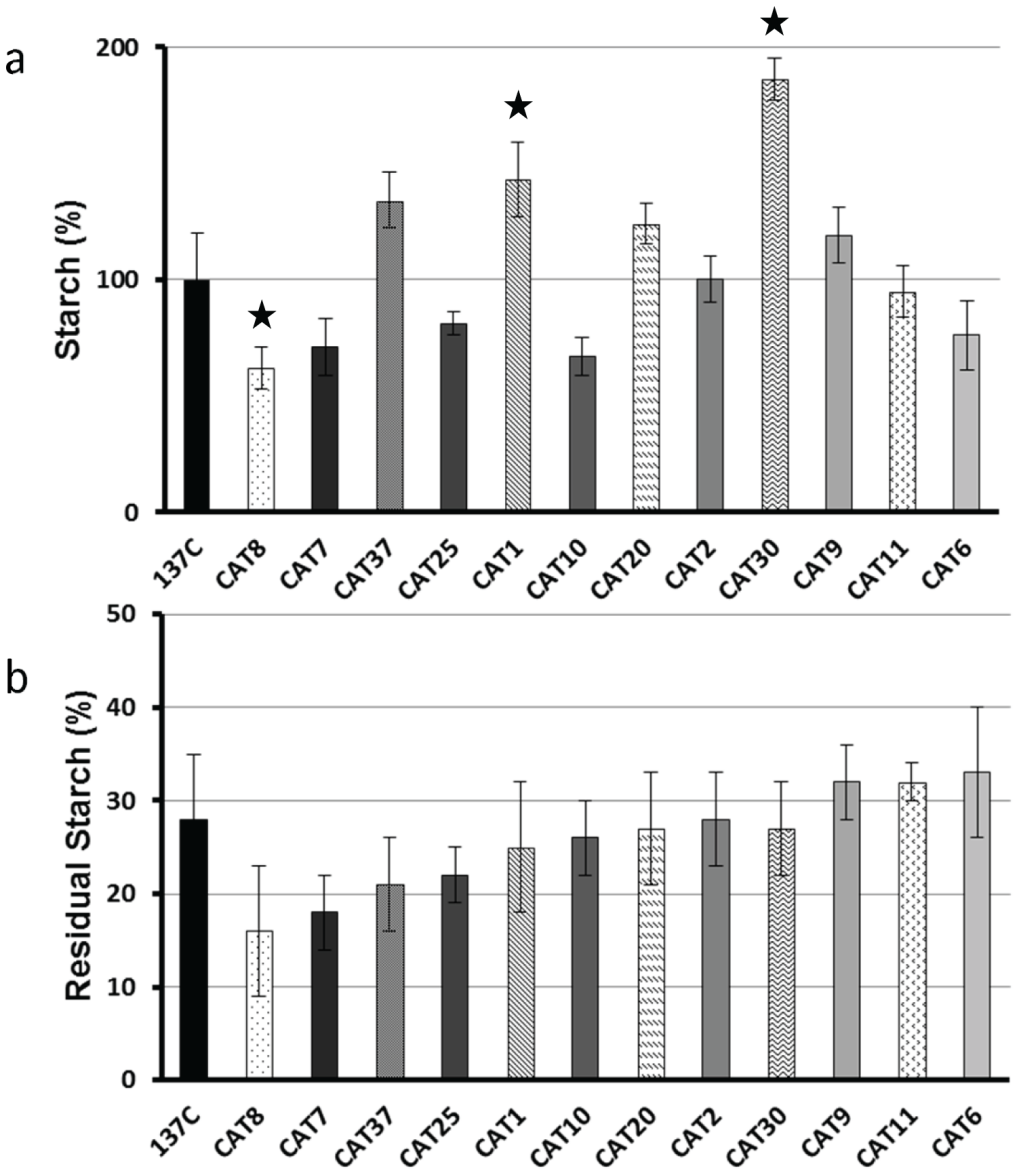
Starch deposition and kinetics of starch mobilization in class 1 mutants. The amount of starch assayed in the strains after 5 days under nitrogen starvation (a) is displayed as a percentage of the value assayed in the wild-type reference strain 137C (21±4 µg per million cells) cultivated under the same conditions. The amount of starch remaining after 1 day of degradation of the same strains (b) is represented as the percentage of the initial amount measured in the corresponding strain. All data correspond to mean ±SE of three independent experiments. Significant differences with the wild-type 137C (p<0.05) are indicated with a star.

**Figure 3:**
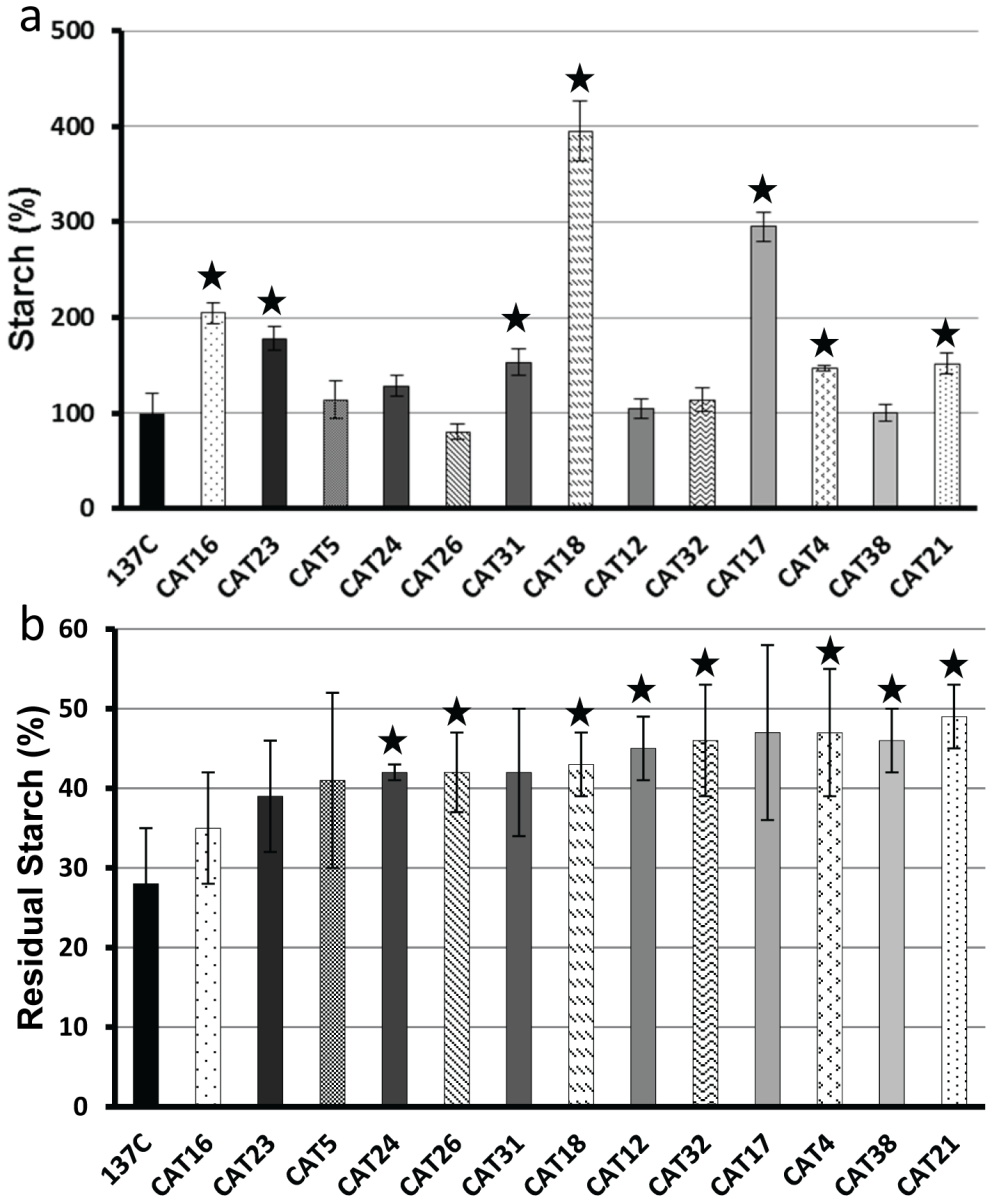
Starch deposition and kinetics of starch mobilization in class 2 mutants. The amount of starch assayed in the strains after 5 days under nitrogen starvation (a) is displayed as a percentage of the value assayed in the wild-type reference strain 137C (21±4 µg per million cells) cultivated under the same conditions. The amount of starch remaining after 1 day of degradation of the same strains (b) is represented as the percentage of the initial amount measured in the corresponding strain. All data correspond to mean ±SE of three independent experiments. Significant differences with the wild-type 137C (p<0.05) are indicated with a star.

**Figure 4:**
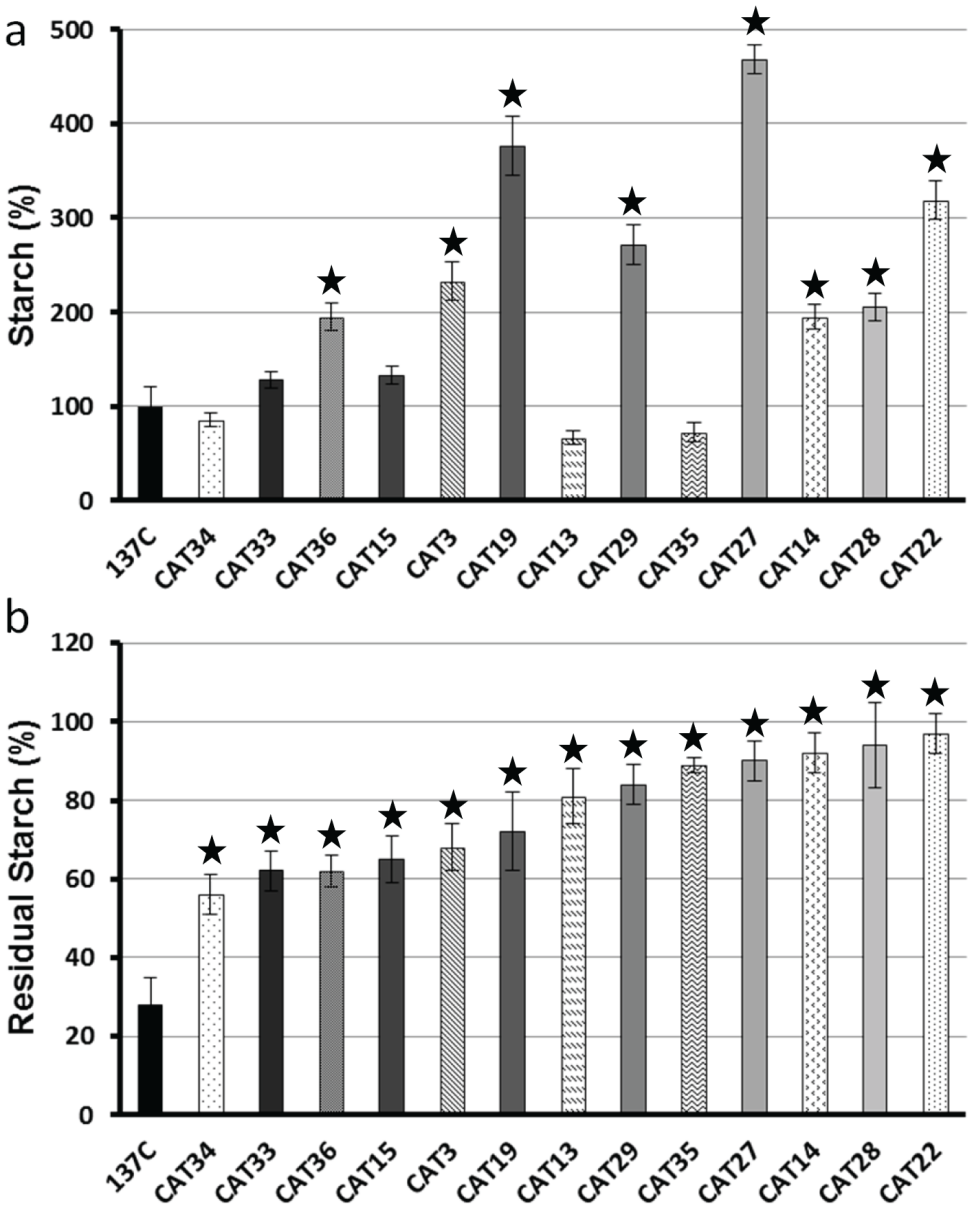
Starch deposition and kinetics of starch mobilization in class 3 mutants. The amount of starch assayed in the strains after 5 days under nitrogen starvation (a) is displayed as a percentage of the value assayed in the wild-type reference strain 137C (21±4 µg per million cells) cultivated under the same conditions. The amount of starch remaining after 1 day of degradation of the same strains (b) is represented as the percentage of the initial amount measured in the corresponding strain. All data correspond to mean ±SE of three independent experiments. Significant differences with the wild-type 137C (p<0.05) are indicated with a star.

Starches accumulated by each of the CAT mutants were assayed after 5 days in liquid TAP-N medium under constant light. The amounts of storage polysaccharide accumulated in all these strains are displayed in figures 2a, 3a and 4a as percentages of the amount assayed in the wild-type. We were expecting that most of our selected mutants would have displayed a starch excess phenotype. This is clearly not the case as 16 of them contain between 64 to 119% of the wild-type value (the wild-type strain 137C contains 21±4 µg of starch per million cells in our standard nitrogen starvation conditions). Fifteen CAT mutants contain a significant higher amount of starch under nitrogen starvation (ranging from 124% to 205%) and only 7 display a clear starch excess phenotype containing more than twice the amount of polysaccharide accumulated by the parental strain (from 233% to 468%). In parallel, we examined the kinetics of starch degradation in each of our strains in liquid cultures. Each strain was used to inoculate one liter of TAP-N liquid medium and grown for 5 days under continuous light. The cells were then transferred to TMP medium (without acetate) and aliquots of the culture were incubated in the dark for 4, 8 and 24 hours. For each time, starch was extracted and assayed in order to determine the amount of polysaccharide broken down. The amounts of residual starch after 24 hours of degradation are displayed in figures 2b, 3b and 4b while the complete kinetics can be found in figure S1. These kinetics experiments allowed us to identify three phenotypic classes in our mutant bank. The first group consisted in strains as efficient as the wild-type with respect to starch mobilization. In fact, after 24 hours darkness, the wild-type 137C strain still contained 28±7% of its initial starch amount as did 12 of our mutants (figure 2b). Those strains which initially displayed a clear and confirmed phenotype during the 2 step screening procedure should be then either considered as false positives or as mutants with conditional defects due to different behavior on solid and liquid medium. However, one of them called CAT37 did display a slow rate of starch mobilization during the first 8 hours of the kinetics and may represent a mutant in the signalization process (figure S1a). The second class of mutants displayed a confirmed unconditional defect in starch mobilization. These 13 strains (figure 3b) contain more residual starch than the wild-type after 24 h of degradation (from 35 to 49% of the initial amount while 137C contains 28%). Finally, the most defective strains (figure 4b) define the third class of mutants in which 13 strains harbor a strong starch degradation defect as they still contained between 56 to 97% of their initial amount of starch after 1 day in darkness. It is of prime importance to notice that for each class of mutants we observed strains containing completely different amounts of starch. Even for the most affected strains, some did contain less starch than the wild-type at the end of the starvation phase. This is clearly the case for example for strains CAT13, CAT34 and CAT35 which accumulate respectively 67, 86 and 73% of the wild-type starch amount but are still unable to degrade their polysaccharides as they still contain 81, 56, and 89% of this starch after 24 h in TMP in the dark while 137C was able to catabolize two thirds of its initial amount in the same time. This result and the fact that strains that seem to degrade efficiently while overaccumulating starch (CAT30 for example, figure 2a) reveal that there is not an obligate link between a starch excess phenotype and a defect in starch catabolism. However, the highest starch excess phenotypes are still found in the class 3 mutants corresponding to those strains which are the most impaired in starch mobilization (figure 4).

**Figure 5:**
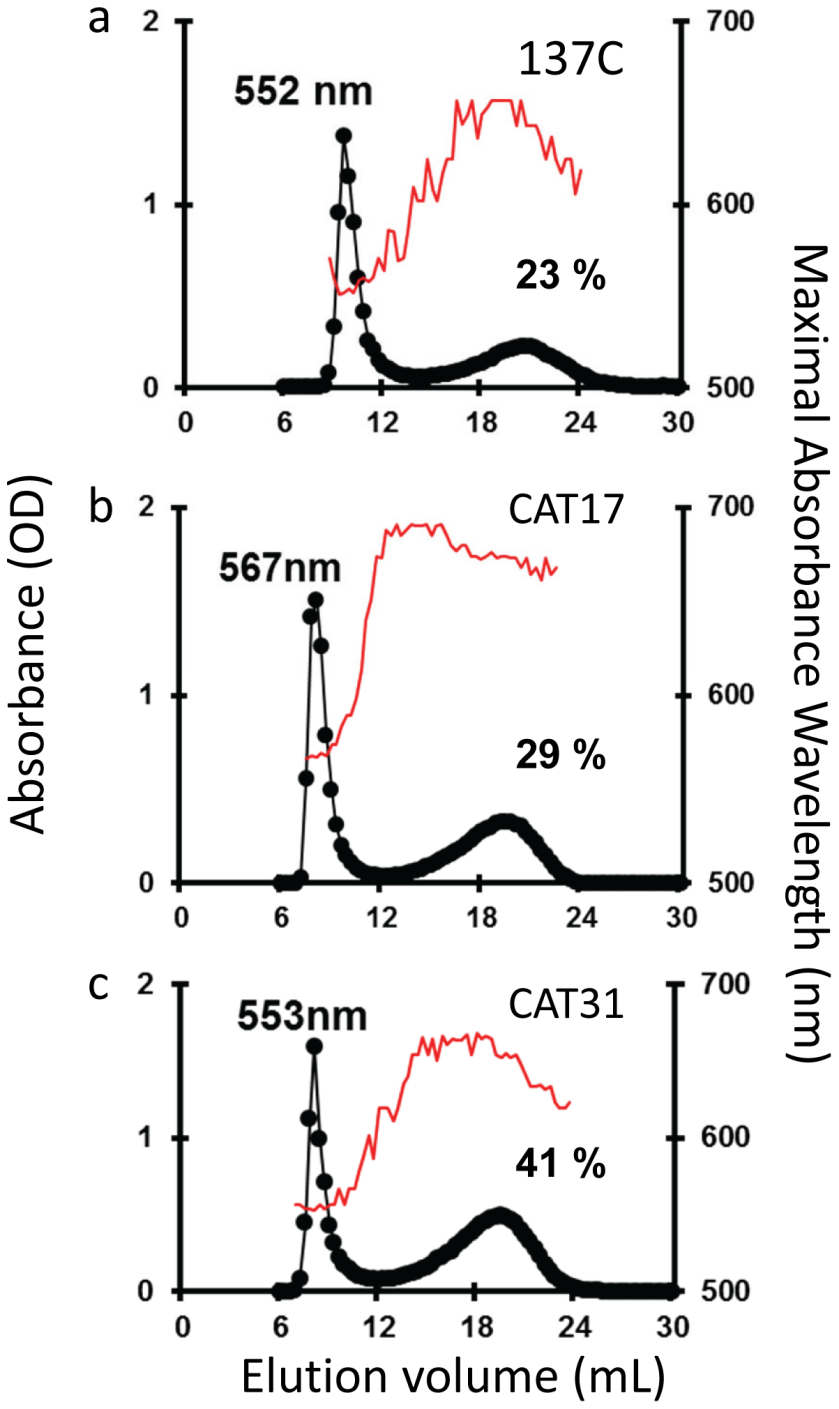
Separation of amylopectin and amylose by CL2B Sepharose chromatography. The optical density (•) was measured for each 0.3-mL fraction at λmax (unbroken thin line). All samples were loaded on the same column setup described by [25]. The wild-type haploid 137C strain starch extracted from nitrogen starved cultures (a) displays both amylopectin and low-molecular weight amylose. Starches from the mutant strains CAT17 and CAT31 are represented in b and c respectively. The amount of amylose (%) and the amylopectin λmax in nanometers are displayed on the corresponding graphs.

Despite that we did not expect mutants impaired in starch degradation to yield a structurally modified polysaccharide, we nevertheless analyzed on CL-2B gel permeation chromatography the amylopectin/amylose ratio and the amylopectin λmax which reflects the structure of this storage polysaccharide component in our CAT mutants (figure 5). The wild-type strain 137C produces a starch composed of 20% of amylose and 80% of amylopectin with a λmax of 550 nm for the latter (Figure 5a). Two of our mutants did harbor significant differences concerning either the amylopectin/amylose ratio or the amylopectin structure. CAT17 produced a starch containing a high λmax amylopectin at 567 nm (Figure 5b) compared to the 552 nm recorded for the wild-type. This increase in λmax is surprisingly not correlated with an amylose enrichment as is usually recorded when amylopectin synthesis is impaired (29% compared to 23% in 137C). On the other hand, the CAT31 starch displayed a high amount of amylose as this component represents 41% of the polysaccharide produced by this strain (figure 5c). In this mutant, the amylopectin structure appears unchanged (λmax 553 nm). At this stage, it seems difficult to explain these modifications and further investigations on these two strains are required to explain the observed phenotypes.

**Figure 6:**
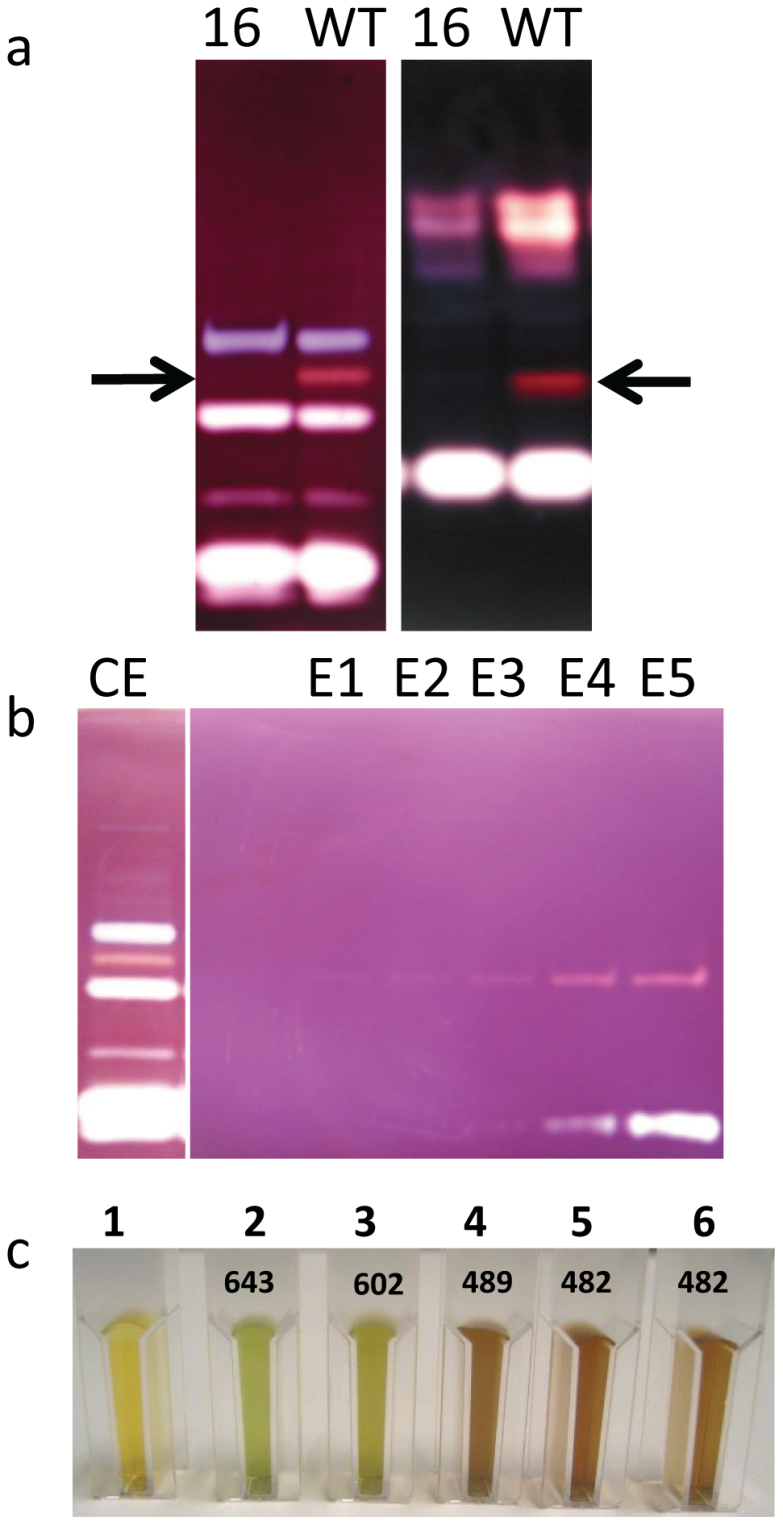
Partial purification of branching enzyme 1 activity. The enzymatic defect in the CAT 16 mutant can be observed through the lack of a pink or a red band (enlighted by arrows) on native (a; left panel) and denaturing (a; right panel) starch zymograms respectively. The lack of contaminating activities in the first elution fractions (E1 to E3) of the amylose column chromatography was assessed on starch denaturing zymogram (b). (c) Interaction of polysaccharides with iodine. Samples 1 to 6 correspond respectively to the iodine alone (1), the interaction of the latter with the unmodified amylose in the absence (2) or in the presence of the MOS in elution buffer (3). The iodine interaction of the polysaccharide modified by the enzyme contained in the 3 elution fractions are displayed in 4, 5 and 6. The values of the λmax of each complex is indicated on the figure in nanometers.

In parallel, we tested the presence of the complete set of hydrolytic activities which are easily visualized thanks to our zymogram techniques in such mutants. In fact, some of our mutants may be defective for an enzymatic activity which is required for starch catabolism and zymograms may allow us to quickly ascertain the absence of one of them. In one of our mutants (CAT16), an activity producing a pink band on denaturing starch containing zymograms is lacking (figure 6a, left panel). This phenotype was confirmed on starch containing zymograms performed under native (non denaturing) conditions (figure 6a, right panel). This pink/red staining of the missing activity in CAT16 is characteristic of an increase in the amount of branches in the starch contained as substrate in the gel. Two kinds of activities can be responsible of such a modification. Branching enzyme will catalyze the introduction of new α-1,6 linked chains in the polysaccharide matrix and β-amylases by shortening the outer chains of starch will produce a so-called beta-limit dextrin which is enriched in branching points compared to the initial substrate found in the gel. To determine the nature of the missing activity in our mutant, we semi purified this activity and determined its precise nature as described below.

### Molecular characterizations of the CAT mutants

In order to confirm the validity of our screening procedure, the mutants displaying a possible defect in starch mobilization (i.e. chiefly the class 2 and 3 mutants) were analyzed through molecular techniques in order to identify the genomic sequences of those regions flanking the insertions. Different strategies including plasmid rescue, inverse PCR and TAIL-PCR were attempted. Even if our efforts resulted in limited success, we were still able to determine in a few of our mutants the identity of the genes disrupted by the exogenous DNA. A summary of the genes identified and of the techniques used for the identification is listed in table 1. We were able to find in our mutant bank several insertions in genes which encoded enzymes previously reported as involved in starch catabolism. This is the case for the CAT3 mutant which is disrupted in a gene encoding the MEX transporter previously characterized in Arabidopsis [18]. We were also able to identify a disruption in a catalytic isoform of β-amylase (CAT4) which had been described as a major component of the transient starch degradation machinery in Arabidopsis [16]. Two additional mutants can be suspected to be defective for components of the catabolic machinery even if these functions had not yet been reported as such. This is the case for the CAT33 mutant which carries a disruption for a protein of unknown function but which contains a CBM20 starch binding module. A disruption in an oligosaccharyl transferase was also detected in CAT37 and may also represent a yet undescribed enzymatic function required for efficient starch degradation. In 4 of our mutants, we were able to identify the integration sites in different kinase activities (table 1). If the link between the starch defect and the absence of these kinase activities should be confirmed, it may unravel precious information concerning the regulation of starch catabolism. However, the phenotype on starch catabolism could also reflect indirect consequences of the absence of a kinase activity since such mutants are known to yield numerous pleiotropic effects. Finally, we detected a defect in a gene described as encoding a flagellar/basal body protein which could explain the starch excess phenotype found in the CAT22 mutant as motility has been previously reported to impact starch accumulation [32]. Moreover, we started backcrosses and genetic analyses of our mutants in order to check the co-segregation between the disruption and the observed phenotype. The complete genetic and biochemical characterization of some of them will be described elsewhere.

**Table 1:** List of mutants whose molecular nature was identified through molecular or biochemical techniques.

Strain	Origin	Identification Technique	Insertion (Phytozome v9.0)
CAT3	Plasmid	Inverse (T1&T2) *BssHII*	Cre12.g486600, Maltose exporter-like protein
CAT4	Plasmid	Inverse (P1&P2) *BssHII*	Cre06.g307150, Beta-amylase
CAT16	Plasmid	Zymograms	Cre06.g289850, Branching enzyme 1
CAT33	PCR	TAIL	Cre02.g091750, CBM20 Starch Binding Domain containing protein
CAT37	PCR	TAIL	Cre07.g330100, Oligosaccharyl transferase STT3 subunit
CAT15	Plasmid	Plasmid rescue *Apa*I	Cre16.g689550, Putative tyrosine kinase
CAT24	Plasmid	Inverse (P1&P2) *EaeI*	Cre06.g307100, ABC1/COQ8 Ser/Thr kinase
CAT26	Plasmid	Inverse (P1&P2) *BsrBI*	Cre02.g107000, Putative protein Kinase
CAT14	Plasmid	Inverse (P1&P2) *BssHII*	Cre06.g266150, Putative kinase
CAT22	Plasmid	Inverse (P1&P2) *BssHII*	Cre07.g338300, Putative flagellar/basal body protein

In this work, we decided to focus on the CAT16 mutant which is the only one in our bank for which we detected a clear enzymatic defect through zymograms.

### The CAT16 mutant is defective for starch branching enzyme 1 and displays a starch catabolism defect

**Figure 7:**
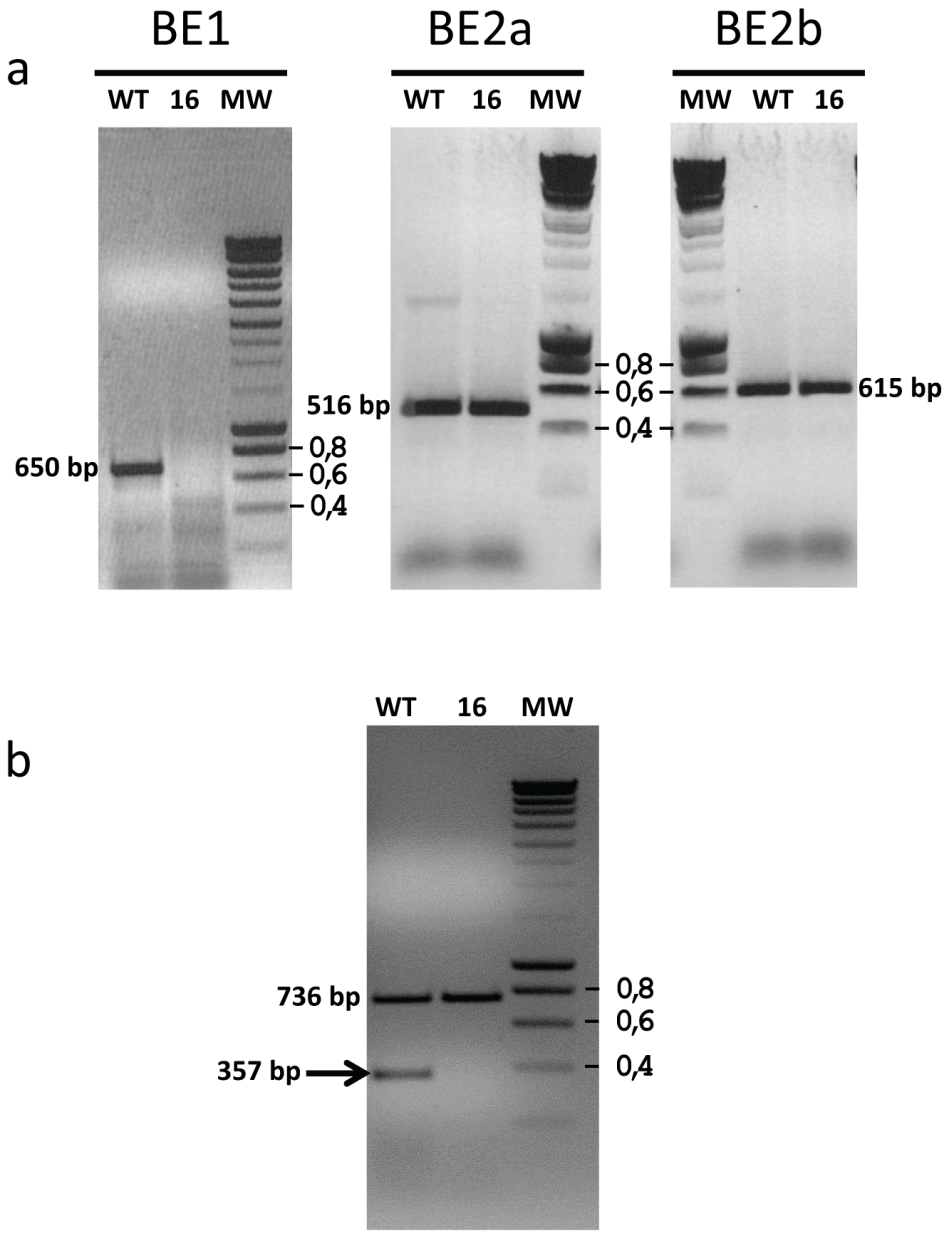
Molecular characterization of the CAT 16 mutant. (a) Amplification of a part of each of the three branching enzyme structural genes was performed on genomic DNA extracted from the wild-type reference 137C (WT) and from the mutant (16). The amplification products sizes for the *BE1*, *BE2a* and *BE2b* structural genes were 650, 516 and 615 bp respectively. (b) RT-PCR analysis performed on total RNAs extracted from the wild-type and the CAT16 mutant allowing the amplification of a 736 bp fragment of the *PHOB* structural gene and a 357 bp fragment of the *BE1* structural gene (indicated by an arrow). Smart ladder (Eurogentec) was used as molecular weight marker (MW), the standard fragments corresponding to 0.4, 0.6 and 0.8 kbp are indicated.

A crude extract from the wild-type strain 137C was used to semi-purify the activity missing in the CAT16 mutant (figure 6a). The wild-type extract was subjected to affinity chromatography on an amylose resin and the bounded proteins were eluted with purification buffer containing increasing amount of malto-oligosaccharides (MOS). Each elution fraction was tested on starch containing zymograms in order to detect fractions containing the pink band forming activity free of any other hydrolytic activity. In 3 elution fractions (E1 to E3 in figure 6b which corresponds to the 3 consecutive elution with the buffer containing 0,5 mg/mL MOS) only the pink band which is missing in CAT16 could be detected on the gel while the next elutions are all contaminated with hydrolytic activities giving a white band on the gel that we already characterized as α-amylase [27]. These fractions were then used to determine the nature of the enzyme activity. To do so, 300 µL of the three fractions were incubated 2 hours in the presence of potato amylose at 30°C. The modified amylose was then stained with iodine (see methods). If the missing activity detected in the CAT16 mutant is of the beta-amylase type, then during the incubation the amylose should be entirely degraded and no interaction with iodine should be recorded. In the case of a branching enzyme activity the greenish color due to the interaction of the long amylose chains with iodine should disappear and a pink to red staining should appear due to the appearance of new branching points. In this case, the λmax of the iodine/polysaccharide complex should decrease due to the reduction in length of the glucose chains [33]. No interaction can be recorded between iodine and the purification buffer used alone (figure 6C, lane 1). A clear interaction of amylose and iodine prior to incubation is observed as revealed by a greenish color with a typical λmax of 643 nm due to the long chains composing the polysaccharide (figure 6C, lane 2). In the presence of 0,5 mg/mL MOS, the λmax of the amylose/iodine complex decreases but remains high at 602 nm showing a light interaction with the glucans that were used to elute the enzymatic activity (figure 6C, lane 3). After incubation with the “pink band” producing activity, the iodine interaction was completely changed as the color switched from green to red and the λmax drastically decreased with values around 480 nm (figure 6C, lanes 4, 5 and 6). This modification reveals that the semi purified activity which is lacking in CAT16 is a branching enzyme activity. To ensure this modification was really due to the “pink band” detected on zymograms, the same purification procedure was used on a crude extract of the CAT16 mutant strain which is lacking this activity (figure S2). As expected, no activity was detected in the elution fractions (figure S2B) and after incubation of these fractions with amylose, no modification of the polysaccharide was detected (figure S2C) revealing the link between the “pink/red” producing enzyme and the branching enzyme activity. As the Chlamydomonas genome contains three kind of branching enzymes, we tried to determine which one was missing in our mutant and if this absence was due to the insertion of the mutagenesis plasmid in the structural gene encoding the enzyme. We then designed three primers couples to amplify a part of the three branching enzyme structural genes from the CAT16 genomic DNA. We were able to amplify a fragment of *BE2a* and *BE2b* structural genes in both 137C and CAT16 while no amplification was obtained with the mutant with the primers designed to amplify a part of the *BE1* gene (figure 7a). As insertional mutagenesis in Chlamydomonas is known to cause deletions, the lack of amplification is a strong argument in favor of an insertion of pSL18 into the *BE1* structural gene. We then tested through RT-PCR the expression of the *BE1* gene. As expected, we were not able to amplify a part of the *BE1* cDNA when using the RNAs extracted from the mutant (figure 7b). These preliminary results may argue in favor of an unsuspected role of branching enzyme 1 in starch catabolism in Chlamydomonas.

## Discussion

While the iodine vapors staining of cell patches has been proved to be an efficient way to selectively detect algal mutants affected in starch biosynthesis and (or) starch structure [26]; the use of this natural interaction had to be tested with respect to starch catabolism. A two-step procedure consisting of the staining of Chlamydomonas cell patches with iodine after massive starch accumulation under the light and a duplicate which is incubated one more day in darkness to trigger degradation was recently used to produce mutants and study the link between polysaccharide degradation and photosystem II-independent hydrogen bioproduction [30]. However, in this study, the mutations were not characterized and it appeared important to ascertain that true starch catabolic mutants could be obtained through this screening procedure. Several physiological processes suspected to be highly regulated impact the amount of polysaccharide remaining after incubation in darkness during the phenotypic screening procedure. First, the cells are incubated 5 days on solid medium without nitrogen which triggers starch deposition. Depending of the strain genotype, the amount of storage polysaccharide accumulated can be different from strain to strain and thereby affect the intensity of the stains. New signals have to be integrated by the cells when starvation is removed and cell patches transferred to darkness. At this stage again, a high amount of residual starch which will impact the stain intensity can reflect not only the presence of a defect for a starch catabolic function but also any mutation influencing the complex machinery responsible for the detection of either the switch to darkness or the starvation status and the equally complex signaling cascades. We then decided to conduct an insertional mutagenesis assay in Chlamydomonas in order to ensure that true starch catabolic mutants could indeed be recovered. Our major goal in this work was then to ascertain that we could recover mutations in genes previously reported or suspected to be selectively involved in starch mobilization. In this case, the effectiveness and at least partial selectivity of the screening procedure would be confirmed thereby allowing a certain level of confidence for the discovery of unsuspected functions.

From 16,000 paromomycin resistant colonies obtained after insertional mutagenesis (13,000 with the linearized pSL18 plasmid and 3,000 with a PCR product corresponding to the resistance cassette) we were able to isolate 38 independent strains which harbor a clear defect for starch mobilization with the two-step iodine screening procedure. Through kinetics of starch mobilization, we were able to show that 12 of these 38 mutants do degrade starch with the same efficiency as the wild-type. Those strains may be either false positive or strains in which the efficiency of starch degradation is not the same on solid and liquid medium. The 26 other mutants were all less effective than the wild-type with respect to starch mobilization and define two different phenotypic classes. The so-called class 2 mutants display a mild phenotype but with a significant starch degradation impairment as these 13 mutants still contain between 35 to 49% of their initial polysaccharide amounts after 1 day of degradation compared to the 28% recorded for the isogenic wild-type. The most defective strains were found in the class 3 for which 13 mutants still contain from 56 to 97% of the initial polysaccharide amounts assayed before degradation. It is worth noting that starch over accumulation and impairment in starch degradation were not linearly linked as overproducers are found in the mutants of class 1 (the one degrading starch with good efficiency) while even in class 3 mutants containing the mutants the most dramatically impaired for starch degradation, some of the strains accumulated nearly wild-type amounts of polysaccharide. These results may reflect the high complexity of the process involved in the regulation of starch deposition and catabolism in Chlamydomonas.

For the 26 strains for which a real defect for starch degradation was confirmed thanks to the kinetics of polysaccharide mobilization in liquid cultures, we used several strategies including the starch structure determination, visualization of the starch hydrolytic activities through the use of zymograms and molecular techniques in order to identify the missing function responsible for the catabolic phenotype.

Even if a defect in starch catabolism is not expected to lead to a modification of starch structure, two of our mutants displayed modification either in the amylopectin/amylose ratio or in the amylopectin structure. The CAT31 mutant contains a high amount of amylose as this starch subfraction represents 41% of the total polysaccharide for only 23% in the wild-type. This increase in amylose is not accompanied by a strong modification of amylopectin which displays the classical λmax value around 550 nm. At the opposite, the CAT17 mutant produces a modified amylopectin with a high λmax at 567 nm but no amylose increase in this polysaccharide can be recorded. As for now, it is difficult to explain these modifications and the link between the modified structure and the defect in starch degradation cannot be easily explained. The identification of the disrupted functions in these strains will possibly yield some understanding on the reasons for these observations.

The molecular experiments including plasmid rescue, inverse PCR and TAIL PCR allowed us to identify several candidates which still need to be validated as responsible of the starch degradation defects. Nevertheless, the detection of a copy of the mutagenesis plasmid in the structural genes encoding the maltose transporter Mex (CAT3) or an isoform of β-amylase (CAT4), both functions already demonstrated to impact efficient starch mobilization, are encouraging with respect to the specificity of our mutant screen. Several disrupted functions which may be related to polysaccharide metabolism were also identified as a starch binding domain containing protein in CAT 33 or an oligosaccharyl transferase in CAT 37. Such oligosaccharyl transferase are known to be involved in protein N-glycosylation in the ER lumen [34]. The effect on starch catabolism if really linked to this insertion may reflect a consequence of the malfunction of important proteins involved in starch catabolism or also be related to the building in a direct or indirect manner of the heteroglycan which plays a central role in maltose breakdown in the cytosol [35]. Four independent insertions in structural genes encoding kinase activities were also recorded in the CAT14, 15, 24 and 26 mutants. However, at this stage we are not sure these mutations are directly responsible for the slower starch degradation observed in these strains.

One single mutation was identified through the use of zymogram techniques allowing visualization of hydrolytic activities. The missing activity in the CAT 16 mutant was identified as a branching enzyme activity. The molecular characterizations allowed us to prove that this strain was defective for branching enzyme 1, an enzyme for which no function in starch metabolism has been reported so far. A recent study performed in maize revealed a slight modification of starch structure in this homologous mutant [36]. The endosperm starch produced by the *sbe1a* mutant was more resistant to α-amylase digestion. During kernel germination, shorter coleoptile lengths and higher residual starch were recorded leading to the hypothesis that less efficient starch utilization was taking place in this mutant. However, at this stage a role of branching enzyme 1 in starch catabolism in Chlamydomonas can only be suggested as this and the other mutants reported in this work have to go through a more detailed and comprehensive characterization. Nevertheless, the recovery of such a “mild” defect in starch catabolism suggests that the iodine screening procedure in Chlamydomonas is quite sensitive and effective in this respect.

The discovery of mutants for Mex or β-amylase which are already known as important starch degradation enzymes reveals the two step iodine phenotypic screen as an efficient tool to isolate novel starch catabolic mutants. We believe that the sensitivity of this mutant screening procedure and the physiological plasticity of the Chlamydomonas model will enable this system to bring further light into the complex machinery responsible for starch mobilization in green plants and algae.

## Methods

### *Chlamydomonas reinhardtii* Strains, Growth Conditions, and Media

The wild-type reference *C. reinhardtii* strains used in this study were 137C (*mt- nit1 nit2*) and 37 (*mt+ ac14 pab2*). All experiments were carried out in continuous light (40 mE m^−2^ s^−1^) in the presence of acetate at 24°C in liquid cultures that were shaken without air or CO2 bubbling. Late-log phase cultures were inoculated at 10^5^ cells mL^−1^ and harvested at 2–3.10^6^ cells mL^−1^. Nitrogen-starved cultures were inoculated at 5.10^5^ cells mL^−1^ and were harvested after 5 days at a final density of 1 to 2.10^6^ cells mL^−1^. Recipes for media can be found in [37]. For starch degradation kinetics experiments, the cells harvested after 5 days under nitrogen starvation were collected in sterile conditions and transferred in TMP (TAP medium without acetate; [37]) liquid medium and incubated in the dark for 4, 8 and 24 hours. Starch was purified from these samples and the amount of remaining polysaccharide was assayed and compared to the initial starch amount assayed before the switch to darkness.

### Determination of Starch Levels, Starch Purification, and Spectral Properties of the Iodine-Starch Complex

A full account of amyloglucosidase assays, starch purification on Percoll gradient, separation of amylose and amylopectin on CL-2B gel permeation chromatography and λmax (maximal absorbance wavelength of the iodine polysaccharide complex) measures can be found in [25].

### Generation of the Mutant Library

The wild-type Chlamydomonas reference strain 137C was transformed with either Eco*RI* linearized pSL18 vector [31] which carries the *AphVIII* gene (aminoglycoside 3′-phosphotransferase from *Streptomyces rimosus*) conferring resistance to paramomycin, or with a 1,9 kb PCR product of this plasmid corresponding to the resistance cassette in order to produce transformants according to the protocol described in [38]. The paromomycin cassette was amplified from the pSL18 plasmid with the primers ParoF (5′-ACCATGATTACGCCAAGCGCGCAA-3′) and ParoR (5′-CTCGACATGCGTTCACTTCCTGTC-3′) using Dynazyme Ext (Finnzymes, Espoo, FI) following the manufacturer recommendation with an annealing temperature at 55°C and an extension time of 2 minutes for 30 cycles.

After transformation, cells were plated on paromomycin supplemented TAP (10 mg/l) plates for the selection of transformant lines.

### Iodine screening of putative starch catabolism mutants

The putative catabolic mutants were screened on the basis of their iodine/starch interaction which reflects the amount of polysaccharide in the cells. Cellular patches (20 µL) of the wild-type reference strain 137C, the already characterized mutants BafJ4 [27] and BafR1 [25] which are respectively defective for isoamylase and granule-bound starch synthase and of putative mutants were incubated on nitrogen free solid medium (TAP-N) for 5 days under continuous light in duplicate. Ten microliters of 0.15 M ammonium chloride were added to each spot of one of the two plates and these Petri dishes were incubated 24 hours in complete darkness in order to trigger starch degradation. Both control and test dishes were exposed to iodine vapor and photographed.

### Zymograms, Partial Purification and characterization of branching enzyme activity

Zymograms in starch containing gels allowing the detection of most starch hydrolases and branching enzymes have been described for both undenatured samples [39] and for denatured enzymes [27]. The semi-purification of the activity generating the pink/red staining band on starch-containing zymograms was performed from 1 liter of exponential phase TAP culture of the reference strain 137C. Algae were disrupted by sonication in purification buffer (10 mM Tris/HCl pH 7.5; 10 mM EDTA; 8 mM DTT). Twenty five mg of total proteins were incubated with an amylose affinity resin (New England Biolabs, Ipswich, MA) for 10 minutes at 4°C. The affinity resin was then washed 3 times with 5 mL of purification buffer and bound proteins were eluted with 3 times one mL of buffer containing increasing concentrations of malto-oligosaccharides (0.5, 1, 2, 5 and 10 mg/mL MOS in purification buffer). Fifty microliters of each elution fraction were subjected to zymogram analysis to detect the elution fractions free of interfering activities (i.e. containing only the pink band). These fractions (300 µL) were incubated for 2 hours at 30°C in the presence of 1 mg of potato amylose (Sigma, St. Louis, MO). The polysaccharides were then collected by ethanol precipitation (1.2 mL 100% ethanol; 13000 g 10 min., 4°C). The dried pellets were then resuspended in 100 µL of 10 mM NaOH and the interaction with iodine was assessed by adding 200 µL of Lugol reagent (0.01% I_2_; 0.1% KI) and 700 µL of distilled water. The absorbance was measured every nanometer between 400 and 700 nm to determine the wavelength of the maximal absorbance of the iodine-polysaccharide complex (λmax) which reflects the polysaccharide structure.

### Nucleic acid Techniques

Total RNA was extracted from Chlamydomonas according to Merendino et al., 2003[40]. RT-PCR analysis was performed on 1 µg of total RNA using the One Step RT-PCR Kit (Qiagen) following the manufacturer recommendation. Amplification was achieved with an annealing temperature of 60°C in the presence of Q-solution with the 4 following primers. The BE1RT5 (5′-GAGAGTCACGACCAGGCTCTG-3′) and BE1RT3 (5′-GAAGCCGTAGTGGTTGTCGAG-3′) allow the amplification of a 357 bp fragment of *BE1* mRNA while PhoBRT5 (5′-GCATGTTCCGCCAGACCA-3′) and PhoBRT3 (5′-TGCAGGAAGCGCCAGTTGA-3′) primers are used to amplify a 736 bp fragment of the *PhoB* mRNA used as an internal control. PCR products [23] were resolved in 2% (w/v) agarose gels.

Total DNA from wild-type and mutant strains was extracted as previously described [41].

The integrity of branching enzymes genes was checked by amplifying parts of the corresponding DNA with Dynazyme Ext (Finnzymes, Espoo, FI) following the manufacturer recommendation with an annealing temperature of 60°C and an extension time of 1 minute for 30 cycles. A 650 bp genomic DNA fragment corresponding to BE1 (XM_001695339) was amplified in the reference wild-type using the BE1 FOR5 (5′-ATGGCTGCGAGGCCGCTTCAG-3′) and BE1 REV3 (5′-ACGACCGCCTACACGCCCTG-3′) primers, while BE2aFor5 (5′-CAATGGCACACCTCCTCCAC-3′) and BE2aRev3 (5′-GTTGAACTGGATCTCGTTCCAG-3′) primers were used to amplify a 516 bp DNA fragment and BE2bFor5 (5′-CTTACTCGCACCAGCAAGCTG-3′) and BE2bRev3 (5′-CTTGAAGGTGTACTGCTTGTC-3′) a 615 bp fragment corresponding respectively to the *BE2a* (XM_001696177) and *BE2b* (XM_001690270) genes.

Thermal asymmetric interlaced PCR and inverse-PCR were used to obtain the DNA flanking the paramomycin cassette or pSL18 insertion sites [42, 43,.44] Homology searches were performed using the BLAST server (http://www.ncbi.nlm.nih.gov/BLAST; [45]), and at Phytozome site (http://www.phytozome.net/search.php?show=blast&method=Org_Creinhardtii). For TAIL-PCR the primers used were the degenerated primer AD2 (5′-NGTCGASWGANAWGAA-3′) and 3 specific primers, TAIL1 (5′-GTGCTCGTTTGTCGCTGAAAGT-3′), TAIL2 (5′-CAAATCAGTCCTGTAGCTTCA-3′), and TAIL3 (5′-ACATACGCACCAATCATGTCA-3′) used respectively for the primary, secondary and tertiary reactions. For inverse PCR, the P1 (5′-CATGTTTGCCCGAACTCGGAG-3′) and P2 (5′-CATTTGCCTGCCTTCACGCATC-3′) or the T1 (5′-TGCATGTAATGGCCAGGCCATG -3′) and T2 (5′-ACTGGCTCACGCACACGCTAAC-3′) primers allowed the amplification of the genomic DNA flanking each side of the unique *EcoR*I site used to linearize the pSL18 plasmid. The cycling parameters for all reactions of TAIL-PCR and inverse PCR are described in Table 2.

**Table 2:** TAIL-PCR and inverse PCR cycling parameters used to isolate flanking DNA from insertional mutants.

		TAIL PCR	
Reaction	Step	Thermal settings	N° of cycles
**Primary**	1	93°C, 2 min; 95°C, 1 min	1
	2	94°C, 1 min; 65°C, 1 min; 72°C, 3 min	7
	3	94°C, 1 min; 25°C, 1 min; ramping to 72°C over 3 min; 72°C, 2.5 min	1
	4	94°C, 30 s; 65°C, 1 min; 72°C, 3 min; 94°C, 30 s; 65°C, 1 min; 72°C, 3 min; 94°C, 30 s; 48°C, 1 min; 72°C, 3 min	15
	5	72°C, 5 min	1
**Secondary**	1	94°C, 30 s; 65°C, 1 min; 72°C, 3 min; 94°C, 30 s; 65°C, 1 min; 72°C, 3 min; 94°C, 30 s; 44°C, 1 min; 72°C, 3 min	15
	2	72°C, 5 min	1
**Tertiary**	1	94°C, 1 min; 50°C, 1 min; 72°C, 3 min	30
	2	72°C, 5 min	
			

		INVERSE PCR	
	Step	Thermal Settings	N° of cycles
	1	95°C, 1 min	1
	2	95°C, 1 min; 55°C, 1 min; 72°C, 3 min	30
	3	72°C, 10 min	1

## Supporting Information

Figure S1 **Kinetics of starch mobilization in the insertional mutants.** The amount of starch measured in the CAT mutant strains is displayed as percentages of the initial content after 4, 8 and 24 h of degradation. The class 1 mutants are presented in a while the class 2 and 3 are shown in b and c respectively. Each bar is mean ±SE of three independent experiments. Significant differences with the wild-type 137C (p<0.05) are indicated with a star.
(TIF)

Figure S2 **Partial purification of branching enzyme 1 activity from the CAT16 mutant crude extract.** The enzymatic defect in the CAT 16 mutant can be observed through the lack of a pink or a red band (enlighted by arrows) on native (a; left panel) and denaturing (a; right panel) starch zymograms respectively. This activity cannot be detected in the first elution fractions (E1 to E3) of the amylose column chromatography as it was the case with the wild-type crude extract as reveled by analysis on starch denaturing zymogram (b). (c) Interaction of polysaccharides with iodine. Samples 1 to 6 correspond respectively to the iodine alone (1), the interaction of the latter with the unmodified amylose in the absence (2) or in the presence of the MOS in elution buffer (3). The iodine interaction of the polysaccharide incubated with the 3 elution fractions (E1 to E3) are displayed in 4, 5 and 6. The values of the λmax of each complex is indicated on the figure in nanometers.
(TIF)
