# Supplementary figures and images for: A Forward Genetic Approach in Chlamydomonas reinhardtii as a Strategy for Exploring Starch Catabolism
Source: PLoS One. 2013 Sep 3;8(9):e74763. doi: 10.1371/journal.pone.0074763 (PMC3760859; doi:10.1371/journal.pone.0074763)

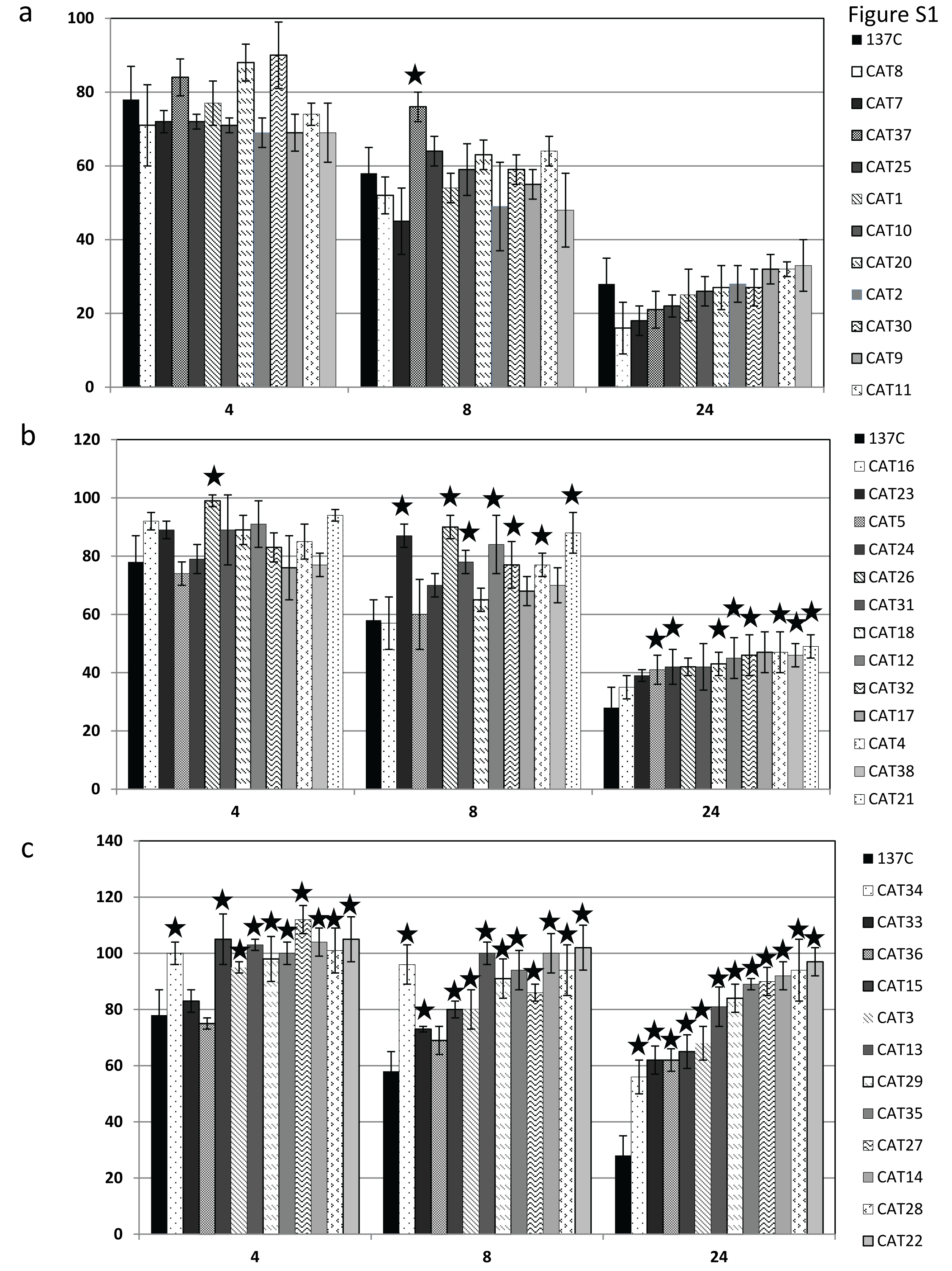

Supplement: Figure S1 — Kinetics of starch mobilization in the insertional mutants. The amount of starch measured in the CAT mutant strains is displayed as percentages of the initial content after 4, 8 and 24 h of degradation. The class 1 mutants are presented in a while the class 2 and 3 are shown in b and c respectively. Each bar is mean ±SE of three independent experiments. Significant differences with the wild-type 137C (p<0.05) are indicated with a star. (TIF) [file pone.0074763.s001.tif]

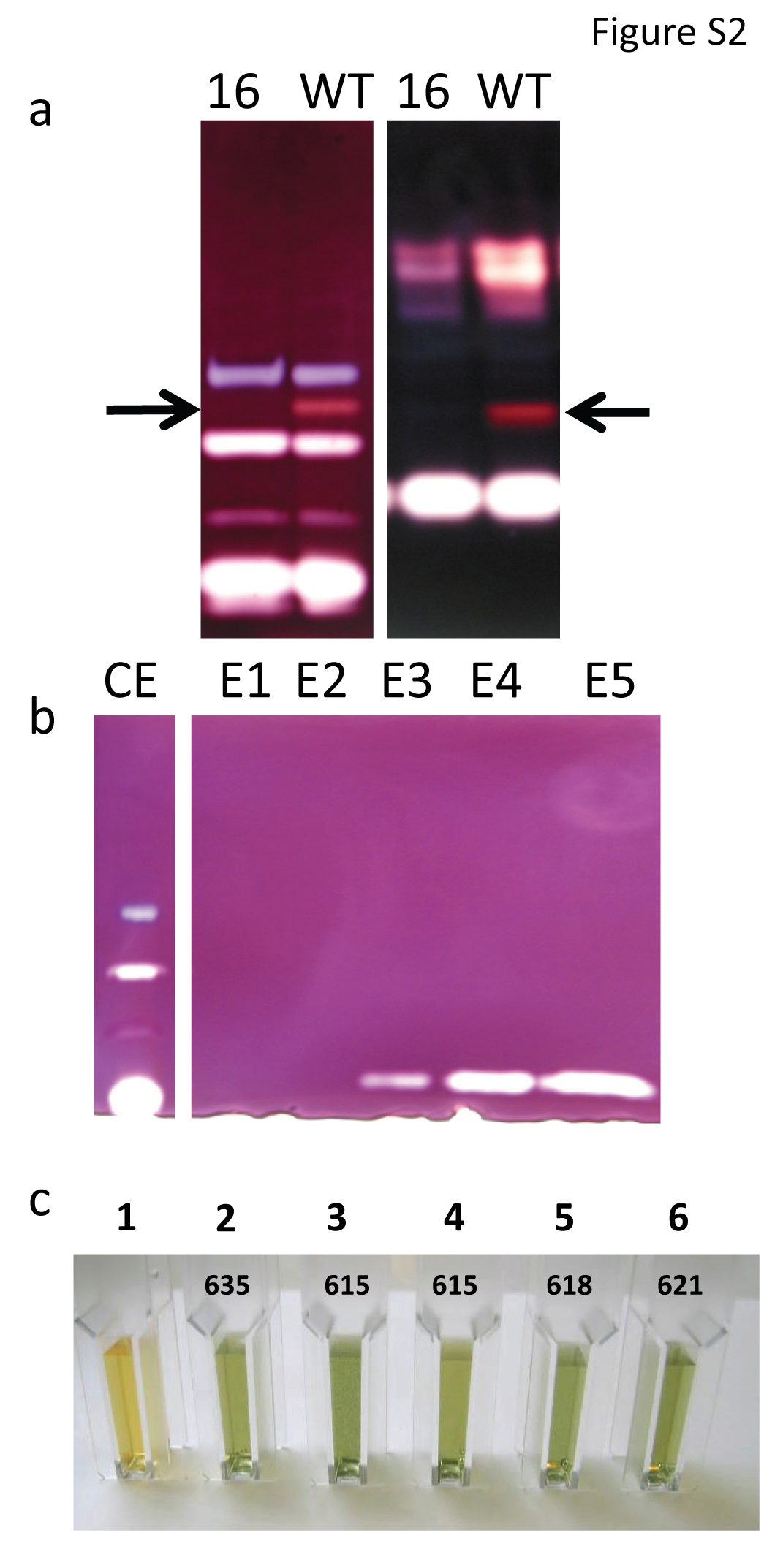

Supplement: Figure S2 — Partial purification of branching enzyme 1 activity from the CAT16 mutant crude extract. The enzymatic defect in the CAT 16 mutant can be observed through the lack of a pink or a red band (enlighted by arrows) on native (a; left panel) and denaturing (a; right panel) starch zymograms respectively. This activity cannot be detected in the first elution fractions (E1 to E3) of the amylose column chromatography as it was the case with the wild-type crude extract as reveled by analysis on starch denaturing zymogram (b). (c) Interaction of polysaccharides with iodine. Samples 1 to 6 correspond respectively to the iodine alone (1), the interaction of the latter with the unmodified amylose in the absence (2) or in the presence of the MOS in elution buffer (3). The iodine interaction of the polysaccharide incubated with the 3 elution fractions (E1 to E3) are displayed in 4, 5 and 6. The values of the λmax of each complex is indicated on the figure in nanometers. (TIF) [file pone.0074763.s002.tif]
